# Cigarette Butt Waste as Material for Phase Inverted Membrane Fabrication Used for Oil/Water Emulsion Separation

**DOI:** 10.3390/polym13121907

**Published:** 2021-06-08

**Authors:** Aris Doyan, Chew Lee Leong, Muhammad Roil Bilad, Kiki Adi Kurnia, Susilawati Susilawati, Saiful Prayogi, Thanitporn Narkkun, Kajornsak Faungnawakij

**Affiliations:** 1 Master of Science Education Program, University of Mataram, Jl. Majapahit No. 62, Mataram 83125, Indonesia; susilawatihambali@unram.ac.id; 2 Physics Education, FKIP, University of Mataram, Jl. Majapahit No. 62, Mataram 83125, Indonesia; 3 Department of Chemical Engineering, Universiti Teknologi PETRONAS, Bandar Seri Iskandar 32610, Malaysia; leongchewlee@gmail.com; 4 Faculty of Applied Science and Technology, Universitas Pendidikan Mandalika (UNDIKMA), Jl. Pemuda No. 59A, Mataram 83126, Indonesia; saifulprayogi@ikipmataram.ac.id; 5 Faculty of Integrated Technologies, Universiti Brunei Darussalam, Jalan Tungku Link, Gadong BE1410, Brunei; 6 Department of Chemistry, Faculty of Mathematics and Natural Sciences, Institut Teknologi Bandung, Bandung 40132, Indonesia; kurnia.kikiadi@gmail.com; 7 National Nanotechnology Center (NANOTEC), National Science and Technology Development Agency (NSTDA), 111 Thailand Science Park, Pathum Thani 12120, Thailand; thanitporn.nar@ncr.nstda.or.th (T.N.); kajornsak@nanotec.or.th (K.F.)

**Keywords:** cellulose acetate, cigarette waste, membrane fabrication, crossflow filtration, oily wastewater, phase inversion

## Abstract

The increasing rate of oil and gas production has contributed to a release of oil/water emulsion or mixtures to the environment, becoming a pressing issue. At the same time, pollution of the toxic cigarette butt has also become a growing concern. This study explored utilization of cigarette butt waste as a source of cellulose acetate-based (CA) polymer to develop a phase inverted membrane for treatment of oil/water emulsion and compare it with commercial polyvinylidene difluoride (PVDF) and polysulfone (PSF). Results show that the CA-based membrane from waste cigarette butt offers an eco-friendly material without compromising the separation efficiency, with a pore size range suitable for oil/water emulsion filtration with the rejection of >94.0%. The CA membrane poses good structural property similar to the established PVDF and PSF membranes with equally asymmetric morphology. It also poses hydrophilicity properties with a contact angle of 74.5°, lower than both PVDF and PSF membranes. The pore size of CA demonstrates that the CA is within the microfiltration range with a mean flow pore size of 0.17 µm. The developed CA membrane shows a promising oil/water emulsion permeability of 180 L m^−2^ h^−1^ bar^−1^ after five filtration cycles. However, it still suffers a high degree of irreversible fouling (>90.0%), suggesting potential future improvements in terms of membrane fouling management. Overall, this study demonstrates a sustainable approach to addressing oil/water emulsion pollution treated CA membrane from cigarette butt waste.

## 1. Introduction

Trillions of cigarette butts are hazardous material deposited annually in the environment [1], and they have been identified as the most littered item worldwide [2]. During the year 2016, 5.7 trillion cigarettes were consumed worldwide, and about 97% of the cigarette filters were composed of cellulose acetate, a modified natural polymer [3]. This figure is expected to increase by 1.6 times in 2025 [4]. The scientific community has been actively seeking economical and sustainable solutions to tackle the cigarette butt waste pollution issue. To date, the alternatives to handle the pollution include degradation, incineration, recycling, and landfilling. Several studies on converting the waste cigarette butts into usable products were made in various fields, mainly in environmental engineering, buildings and infrastructures, energy storage devices, insecticide, and metallurgical industry [5,6]. The analysis of the potential recycling cigarette butt waste in environmental engineering applications corresponds to about 14.0% of all the possible applications [5]. The utilizations of cigarette butt waste have been mostly focused on buildings and structure applications.

A large volume of oily wastewater is emitted into the environment. The oily wastewater is mainly generated from industries such as petrochemical, petroleum refineries, food manufacturing, and metallurgical [7]. Oily wastewater types include unstable oil/water emulsion (or simply oil/water mixture), stable oil/water emulsion, and free-floating oil [8]. The continuous and increasing discharge of oily wastewater can severely endanger the ecosystem and pollute the environment. Without proper treatment, emulsified oily wastewater can contaminate the groundwater resources in which drinking water and agricultural production are affected [7].

Conventional methods (flotation and coagulation) for the treatment of stable oil/water emulsions are less effective in handling micron-sized emulsion droplets and finely dispersed oil particles [9]. The membrane-based process is seen as one of the emerging methods for treating oil/water emulsion wastewater that has been shown effective in handling low concentration of oil (<1000 ppm) in water [10,11,12]. It outstands the conventional separation techniques for simplicity, continuous, faster, and cost-effectiveness due to their low energy consumption.

The main component of cigarette butt is cellulose acetate (CA) [13], suitable to be converted into polymeric membrane for liquid-based filtration [14]. Cellulose acetate is a cellulose derivative, which possesses good transparency and mechanical strength. Almost 90% of cigarettes are manufactured with cellulose acetate filter tips (cigarette butt) [15]. Cigarette butts contain up to 96.0% of cellulose acetate that can be used to form the membrane material, as explored in this study. This way, the circular economy concept can be implemented by providing the opportunity to use cigarette butt waste into economically attractive and usable products [16,17].

A recent study showed that cellulose acetate from waste cigarette butt can be used as raw material for the fabrication of nanofiber membrane [18]. The nanofiber achieved 99.9% of oil droplet separation efficiency when used to treat oil-in-water mixtures. The oil/water mixture treated in this work was a less challenging feed of an oil/water mixture. A more challenging feed in the form of oil/water emulsion separation has not been addressed yet. Electrospun nanofiber membranes are notable for their superiority, high efficiency, simplicity, and low cost [19]. Despite that, one of the critical limitations of the electrospun nanofiber membranes is their weak mechanical strength. They cannot be used as a standalone system without an additional supporting layer and/or post-treatment, normally in the form of non-woven membranes [20,21]. Moreover, the electrospinning process is relatively slow and requires a longer time to fabricate a membrane. The standard fabrication time for a sheet of nanofiber net in a lab-scale setup takes up to 100 h. Nonetheless, little attention has been given to other types of membrane fabrication methods to develop CA-based membranes from waste cigarette butts. Therefore, this study explored the application of cigarette butt as the polymer-based material for membrane fabrication through the established phase inversion method [22] for treating the challenging oil/water emulsion separation. Numerous researches have been conducted to improvise the properties of the membrane from an established polymer, such as polyvinylidene difluoride (PVDF) and polysulfone (PSF) through modification of fabrication parameters and post-treatments [23,24].

In this study, we explore the utilization of waste cigarette butt as material for the fabrication of phase inverted membranes. The resulting membrane was compared with the phase inverted membrane fabricated from commercial PSF and PVDF polymers, both polymers are the most used in the commercial membranes for low-pressure filtration (i.e., membrane bioreactor) [25]. After fabrication, all the membranes were characterized in terms of mean flow pore size, surface contact angle, morphology, and clean water permeability. Finally, the filtration performance of the membranes was evaluated for filtration of synthetic oil/water emulsion. This approach epitomized a circular economy in which cigarette butt waste was converted into another valuable material for protecting nature when applied for treating wastewater.

## 2. Materials and Methods

### 2.1. Materials

The dope solution compositions of the three membranes used in this study are summarized in Table 1. The detail on fabrication and filtration of the plain PVDF and the PSF (Mw = 35,000 g/mol, Sigma Aldrich, St Louis, MO, USA) membranes are available in our earlier reports [10,12]. For fabrication of CA-based membrane, discarded cigarette butts were collected from public smoking areas. There was no specific criterion for pre-screening of the cigarette butts collection. The collected cigarette butts were first cleaned physically by removing any remaining tobacco, wrapping papers, and burnt tips. The cigarette butts went through several cleaning cycles, and each cycle consists of immersing and stirring the butts in boiling water. They were dried thoroughly at 60 °C in an air-circulating oven overnight to remove the moisture content. The cleaned cigarette butts were dispersed in *N*,*N*-dimethylformamide (DMF, Sigma-Aldrich, St Louis, MO, USA) solvent and cast atop a stainless steel mesh (37.0 µm, Guangzhou, China) to provide mechanical strength.

The stabilized oil/water emulsion was synthesized according to an earlier work [26] using crude oil (obtained from a crude oil well in Malaysia), distilled water, and sodium dodecyl sulfate (SDS, 98% purity, Sigma-Aldrich, St Louis, MO, USA). The SDS-to-oil ratio of 1:99 (*w*/*w*) was mixed in water to obtain 1000 ppm stabilized emulsion via mechanical agitation at a stirring rate of 3500 rpm for 24 h. A small volume of feed samples was subsequently analyzed to map the oil droplet size distribution. The sizes of the droplets were in multi-modals distribution with peaks at 0.25, 0.9, and 4.0 µm.

### 2.2. Membrane Preparation

**Figure 1:**
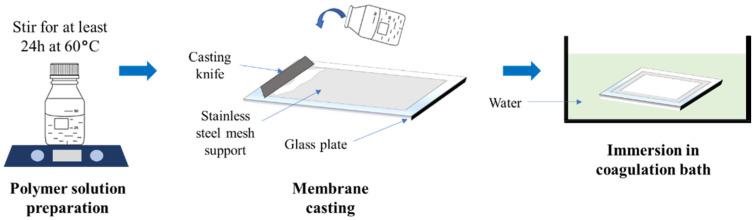
An illustration of phase inversion method for membrane fabrication applied in the present study.

For the preparation of CA-based membrane, the dope was prepared by dispersing 10 wt% of the cleaned cigarette butt in a corresponding amount of DMF without any additive (Table 1). The mixture was stirred for 24 h at 60 °C to ensure the formation of a homogeneous solution. The solution was degassed for several hours to release the entrapped air bubbles before being used for membrane fabrication. The CA membrane was synthesized via the phase inversion method with stainless steel mesh as the support according to the method illustrated in Figure 1. The dope solution was poured on top of a flat stainless-steel mesh placed on the glass plate. The dope solution was cast over the stainless steel mesh using a doctor blade with a wet thickness of 330 µm to form a thin film. Subsequently, the casted film and the glass plate were directly immersed in the non-solvent bath containing deionized water to undergo the phase inversion. The resulting CA membrane was soaked in deionized water until further use.

### 2.3. Membrane Filtration Setup

The filtration system was operated under full recycling mode by constantly returning the permeate to the feed solution after the volume was periodically (of every 10 min) measured. The setup was used to analyze the membrane filtration performance in treating synthetic oil/water emulsion. A peristaltic pump was used to provide a constant transmembrane pressure of 0.2 bar, while keeping the feed flowing through the system at a linear velocity of 13.4 cm s^−1^. The prepared membrane with an effective area of 36.5 cm^2^ was placed in between spacers in a lab-made filtration cell. The filtration was first conducted using deionized water to determine the clean water permeability of the membrane. Each filtration test was conducted for 60 min, in which a queasy steady-state permeability was obtained.

The filtration flux ($J_{s}$, L m^−2^ h^−1^) and permeability (L, L m^−2^ h^−1^ bar^−1^) were calculated using Equations (1) and (2), respectively:

(1) $$J_{s}=\frac{∆V}{A_{s}∆t}$$

(2) $$L=\frac{J_{s}}{∆P}$$

where Δ*V* is the volume of the collected permeate (*L*), *A_s_* is the effective membrane area (m^2^), Δ*P* is the transmembrane pressure (0.2 bar), and Δ*t* is the filtration time (h).

### 2.4. Membrane Characterization

The microstructures, cross-section, and surface morphology images of the resulting membrane were processed using a scanning electron microscope (SEM, Zeiss Evo, Germany). The samples were coated using gold to enhance the conductivity for obtaining good images. The pore size distribution of the membranes was determined using a capillary flow porometer (CFP, Porolux 1000, Berlin, Germany). The energy-dispersive X-ray spectroscopy (EDS) was used to define the elemental composition near the surface of the membrane samples. The hydrophilicity of the membrane surface was determined by the static contact angle using a goniometer (Ramé-Hart 260, Succasunna, NJ, USA). The chemical bonds of the CA membrane sample were identified using the Fourier transform infrared spectrometer (FT-IR, Frontier 01 Perkin Elmer) in the spectra wavenumber range of 400 to 4000 cm^−1^. The concentration of oil content in the feed before and after the filtration tests were studied using a UV-VIS spectrometer (Shimadzu UV-2600, Kyoto, Japan) at a wavelength of 223 nm.

### 2.5. Membrane Fouling Identification

Before obtaining the clean water permeability, membrane compaction was performed for 60 min. The permeability was measured as the average value of the next 30 min. After measuring the clean water permeability, the filtration of oil/water emulsion feed was conducted for five cycles. Each cycle comprised of 30 min filtration, followed by 5 min of membrane flushing with deionized water. From the five filtration cycles, different types of fouling parameters were identified. The total fouling (TF, %), reversible (RF, %), and irreversible fouling (IR, %) of the membrane were determined using Equations (3)–(5), respectively:

(3) $$TF_{n}=\frac{L_{o}-L_{n}}{L_{o}}$$

(4) $$RF_{n}=\frac{L_{o\left(n\right)}-L_{o\left(n-1\right)}}{L_{n}}$$

(5) $$IR_{n}=\frac{L_{n}-L_{o\left(n\right)}}{L_{n}}$$

where *n* is the number of filtration cycle, $L_{o}$ is the clean water permeability at the beginning of the filtration, $L_{n}$ is the average permeability at cycle *n*, $L_{o\left(n\right)}$ is the permeability of clean water at cycle *n*, $\mathrm{and}L_{o\left(n-1\right)}$ is the permeability of oil/water emulsion filtration at cycle *n* − 1.

## 3. Results and Discussion

### 3.1. Surface and Cross-Section Morphologies

**Figure 2:**
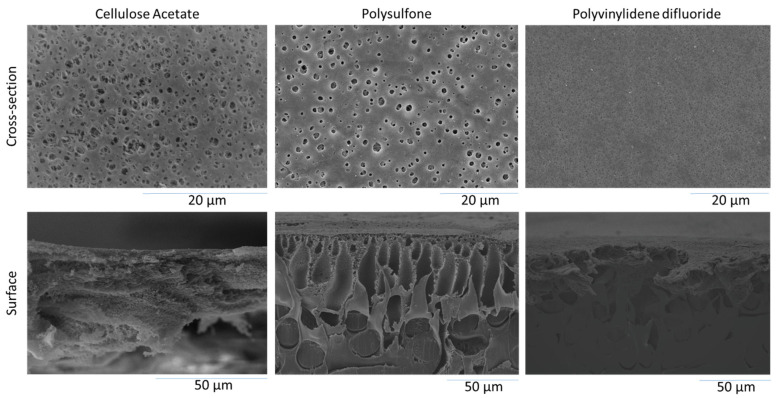
Surface and cross-section SEM images of the membrane samples.

Figure 2 shows the morphological structure of the developed CA, PSF, and PVDF membranes. Based on the top surface SEM images, all the samples pose visible surface pores homogeneously distributed. They show the typical morphology of membranes prepared by non-solvent induced phase separation. Most importantly, despite being prepared from waste cigarette butt, the CA membrane also poses a good surface property such as the one prepared from the commercial PVDF and PSF polymer, typically used for membranes fabrication. The finding on the microstructure suggests the potential of a waste cigarette butt for membrane fabrication, which can be applied for oil/water emulsion filtration. The surface pores are within a size range far below most of the oil droplets presented in the oil/water emulsion feed used in this study.

The cross-section images of all the membranes show equally asymmetrical morphology, a typical structure of membranes prepared from non-solvent induced phase separation under instantaneous demixing [27], in which a dense surface morphology is supported by a more porous structure underneath. The large surface pores of the CA membrane are all within the microfiltration range, which also suggests the instantaneous demixing phase separation mechanism. A recent report on the fabrication of CA membrane from the commercial CA polymer showed symmetric morphology since it was prepared from different solvent/nonsolvent systems and different polymer concentrations [14]. A detailed discussion on the relationships between the polymer/solvent/nonsolvent system can be found elsewhere [14,24,28]. The finding suggests that irrespective of the source (i.e., waste cigarette butt), the CA membrane could be prepared using the phase inversion method resulting in the reliable membrane effectively being used for filtration, as demonstrated in Section 3.7.

### 3.2. Membrane Pore Size and Distribution

**Figure 3:**
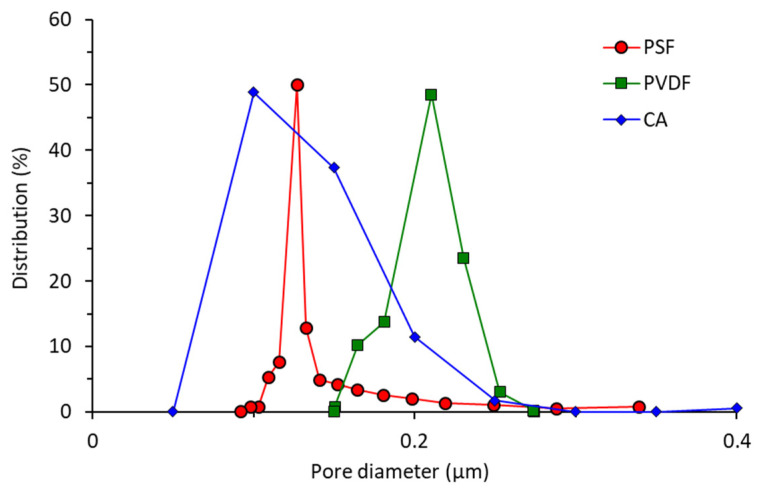
The pore size distribution of the developed cellulose acetate (CA), polysulfone (PSF), and polyvinylidene difluoride (PVDF) membranes.

Figure 3 shows the pore size distribution of the three membrane samples evaluated using a CFP. The *y*-axis of the figure shows the actual distribution of pore of certain size, not the frequency distribution found in a typical histogram. The pore distribution of all membrane samples skews to the left indicating higher populations of smaller pores. The cigarette butt-based CA membrane poses a high pore size population at around 0.10–0.15 µm. The pore size range of the cigarette butt-based CA membrane is suitable for handling the oil/water emulsion since the pores theoretically could retain emulsion droplets with sizes larger than the membrane pore sizes. The sizes of the oil droplets in emulsion are normally in the range of 0.1 to 10 µm [29]. Most of the oil droplets can be effectively removed with a membrane of pore size in the range of 2 to 100 nm. The membrane works based on the size exclusion theory, in which the membrane material rejects particles larger than the pore size. Higher mean flow pore sizes are shown by the PSF and PVDF membranes at 0.127 and 0.210 µm, respectively. It was reported that the typical commercial microfiltration CA-based membrane has a pore size of 0.470 µm [30], most likely due to some differences in fabrication parameters. Indeed, further exploration can still be done to fine-tune the properties of a cigarette butt CA-based membrane according to the required specifications, as suggested elsewhere [31,32,33].

**Figure 4:**
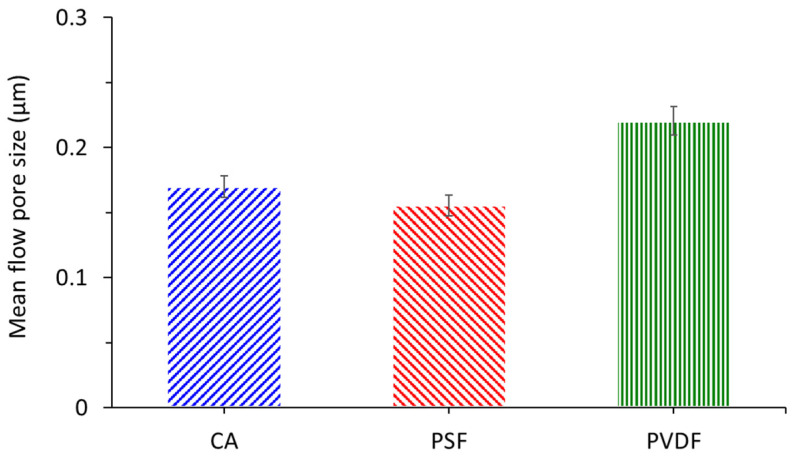
The mean pore size distribution of the cellulose acetate (CA), polysulfone (PSF), and polyvinylidene difluoride (PVDF) membranes.

Figure 4 depicts the mean pore size distribution of CA, PSF, and plain PVDF analyzed with CFP. The CFP test accurately captures the pore size across the thickness and the size distribution shown in Figure 3. It shows that the plain PVDF membrane exhibits the largest mean flow pore size of 0.2206 µm in comparison to CA and PSF, with a mean flow pore size of 0.17 and 0.1556 µm, respectively. The SEM images of the plain PVDF membrane show poor surface pore visibility. Figure 4 shows that the pore size of the membranes is comparable and all are expected to effectively retain oil droplets in the oil/water emulsion feeds. In addition to the mean flow pore size and pore size distribution, the specific number of pore per unit of membrane surface is also important to govern the permeability and can distinguish the throughput of membranes despite having a similar pore size and distribution.

### 3.3. Surface Contact Angle

**Figure 5:**
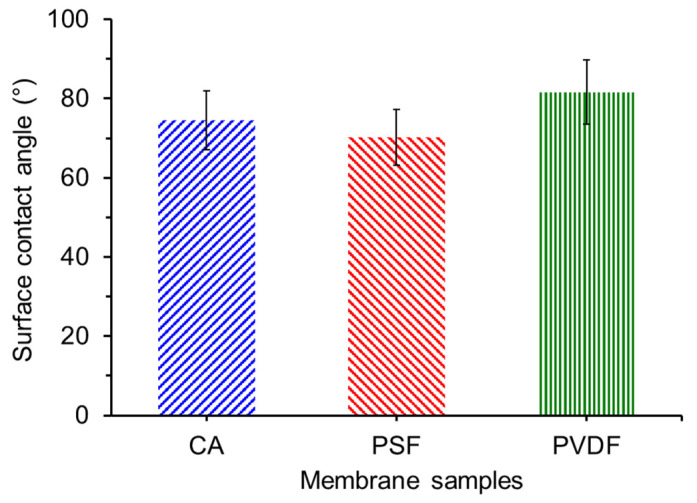
Static contact angle of the developed cellulose acetate (CA), polysulfone (PSF), and polyvinylidene difluoride (PVDF) membranes.

Figure 5 shows the static water contact angle for the three membrane samples used in this study. The static water contact angle is essential in determining the permeability and fouling properties of a membrane. A membrane is considered hydrophilic when the contact angle falls between 0 to 90°. Membranes with hydrophilic properties are ideal in the oil/water emulsion treatment when water is the component that is permeating through the membrane pore and vice versa [34,35]. The hydrophilic surface attracts water by creating a hydration layer and prevents oil droplet interaction with the membrane surface, hence improving oil droplet rejection [36]. As shown in Figure 5, the PVDF membrane demonstrates the most hydrophobic characteristic with a water contact angle of 81.59°, attributed to the low polymer surface free energy [37]. This is followed by CA and PSF membranes with the surface water contact angles of 74.5 and 70.23°, respectively. The surface water contact angle of plain CA membrane from commercial polymers in this study is within the range reported earlier of 50–60° [38,39,40], which can be attributed to the variation surface structure and fabrication parameters and possibly due to the presence of impurities that can be further investigated as the follow-up study. These findings are encouraging and show that a CA membrane based from cigarette butt waste potentially possess a high clean water permeability and good anti-fouling property, at least when compared with the PVDF and PSF membranes samples used as a reference in this study.

### 3.4. Fourier Transform Infrared

**Figure 6:**
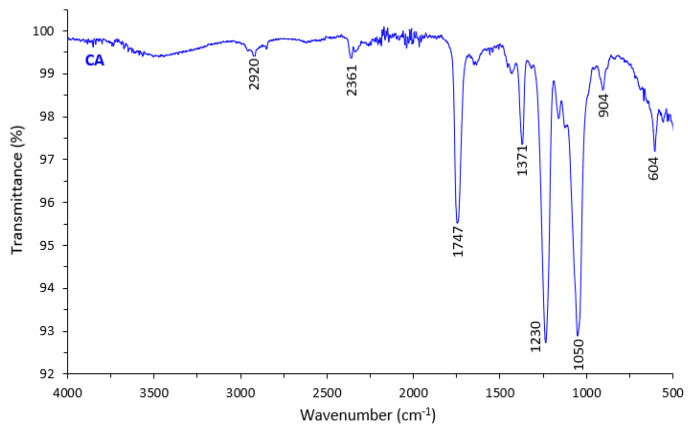
FT-IR spectra of the cellulose acetate membrane.

The FT-IR spectra in Figure 6 depicts the chemical composition of the prepared cigarette butt-based CA membrane. The FT-IR spectrum of CA shows a peak absorption band at 1747, 1230, and 1050 cm^−1^ which is assigned to the C=O carbonyl stretching, C–O stretching, and CO–O–CO stretching. The peaks at 1371 and 2920 cm^−1^ are attributed to the C–O group and aliphatic group (C–H), respectively. Additionally, the broad peak at around 3500 cm^−1^ represents the O–H group. Similar findings were reported by Liu et al. that attributed the presence of carbonyl stretching, symmetric, and asymmetric stretching vibrations of C–O–C, respectively, in the nanofiber membrane from waste cigarette butt [18].

The spectra shown in Figure 6 resemble the one obtained for the phase inverted membrane prepared from the commercial CA polymer [40,41]. The presence of impurities is hardly seen from the spectra, indicating that the spectra associated with them might be overlapping with the spectra associated with CA. Visually, the presence of impurities could be seen from the grey color of the cigarette butt CA-based membrane. The presence of impurities might affect the resulting membrane properties (i.e., higher water contact angle) and the purification process is thus recommended as the follow-up studies. Polymer purification was shown effective in improving the structure and performance of the resulting membranes [42].

### 3.5. Energy Dispersive X-ray Spectroscopy

Table 2 shows the distribution of elemental composition for CA, PSF, and PVDF membranes obtained from EDS mapping. It is observed that the oxygen originating from the hydroxyl group in CA has the highest composition at 48.2%. It is slightly higher than the one obtained from X-ray photoelectron spectroscopy of 42.0% obtained elsewhere [40]. This result indicated the presence of hydrophilic functional groups in the CA membrane, which justifies that the CA membrane has higher hydrophilicity properties than the PSF and the PVDF membranes. The presence of carbon and oxygen is supported by the FT-IR analysis. In contrast, the static contact angle measurement suggests that the PVDF membrane demonstrates the most hydrophilic characteristic with a contact angle of water of 81.59°. The abundance of oxygen element in the CA membrane can further be explored to enhance the surface hydrophilicity.

### 3.6. Clean Water Permeability

**Figure 7:**
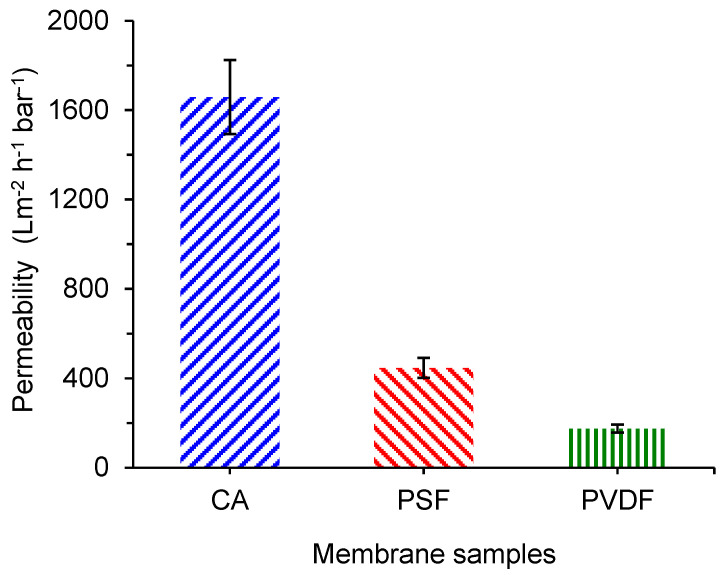
Clean water permeability of the cellulose acetate (CA), polysulfone (PSF), and polyvinylidene difluoride (PVDF) membranes.

Figure 7 shows that the CA membrane outperforms the rest in filtration performance by having the highest permeability compared to PSF and PVDF membranes. Clean water permeability involves the passage of water molecules through the membrane under crossflow filtration. The CA membrane showed that the water permeability of 1658 L m^−2^ h^−1^ bar^−1^ is significantly higher than the PSF and PVDF membranes clean water permeability of 446 and 175 L m^−2^ h^−1^ bar^−1^, respectively.

When considering the pore size and distribution of the three membrane samples evaluated in this study, the significantly high permeability shown by the CA membrane can be ascribed by their low surface water contact angle (Figure 5) combined with the higher surface pore population. Some membranes can show a similar pore size and distribution but differ in pore number, as detailed in an earlier report [43]. When evaluating the surface SEM image in Figure 2, it can be seen that the CA membrane’s surface pores are highly populated compared to the rest.

### 3.7. Filtration Performance

**Figure 8:**
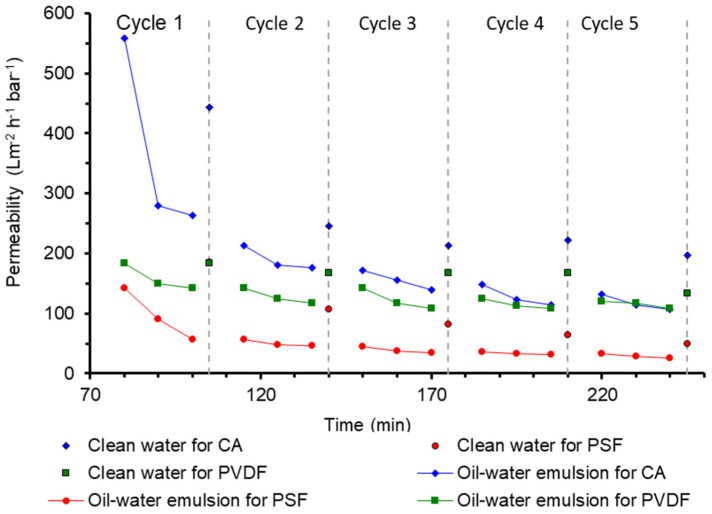
The permeability of the cellulose acetate (CA), polysulfone (PSF), and polyvinylidene difluoride (PVDF) membranes for five cycles in 30 min of oil/water emulsion and 5 min in clean water as a function of filtration time.

Figure 8 shows that the CA poses the highest oil/water emulsion permeability for the first 50 min of filtration, maintained at a value of 180 L m^−2^ h^−1^ bar^−1^ at the end of the subsequent filtration cycles. The high performance of the CA membrane can be attributed to the high oxygen content in the membrane that imposes surface hydrophilicity which is beneficial for repelling deposited oil droplets when treating the oil/water emulsion and forming a hydration layer on the membrane surface [36]. The membrane surface has high surface porosity (from a high number of the surface pore), as shown on the SEM images in Figure 2, which could offer a better oil/water emulsion permeability than the PSF and the PVDF membranes. The clean water flushing introduced at each filtration cycle helps improve the permeability of the membrane and remove the oily foulant and reduce the fouling effect on the membrane. It can be observed that the water flushing at cycle 2, 3, 4, and 5 improve the subsequent permeability of the membrane in oil/water emulsion. However, the water flushing in cycle 1 does not exhibit an increase of permeability. This may occur due to the strong oil adhesion on the membrane surface that has caused the emulsion permeability to dramatically decrease.

In another study, the permeability of the oil/water emulsion for the commercial CA membrane in the first cycle is 1900 Lm^−2^ h^−1^ bar^−1^. After the first flushing, the permeability decreased significantly to 370 Lm^−2^ h^−1^ bar^−1^, following the third cycle of 90 Lm^−2^ h^−1^ bar^−1^. The subsequent cycles show no permeability, which demonstrated that the oil particles have wholly clogged the membrane pores suggesting that severe membrane fouling also happened to a plain CA membrane made from commercial polymer [30]. Although the commercial CA membrane has a high permeability at the initial phase, it is worth noting that the permeability had a steep decrease. When compared to the CA membrane from cigarette waste, the developed membrane exhibited a relatively slow decrease in the whole five cycles. This constitutes an interesting phenomenon as the developed CA is made of cigarette butt waste. A further comparison with PSF and PVDF membranes optimized for oil/water emulsion filtration was reported earlier [10,42]. The permeability is comparable with the plain CA membranes developed from cigarette butt waste reported in the present study. It suggests that the CA-based membrane from cigarette butt, can further be developed to enhance its filtration performance via fabrication parameter optimization or surface modifications.

### 3.8. Rejection Performance

**Figure 9:**
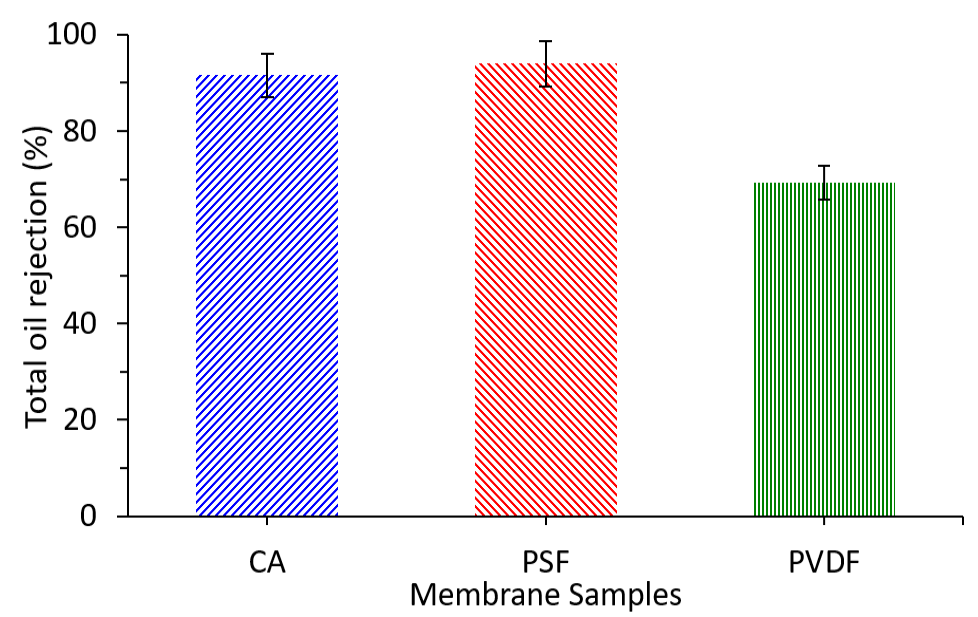
The oil rejection of PW filtration using the cellulose acetate (CA), polysulfone (PSF), and polyvinylidene difluoride (PVDF) membranes.

To evaluate the oil separation efficiency, the oil rejection performances of the CA membrane were evaluated and compared with PSF and PVDF membranes (Figure 9). The CA membrane exhibits an excellent total oil rejection of 91.5%. This shows that the CA membrane developed from cigarette waste is comparable to the established PSF membrane that has achieved the rejection efficiency of 94.0%. In addition, CA could be a promising candidate in achieving a large-scale separation of oil/water emulsion for its greater oil rejection than the PVDF membrane. A study by Liu et al. found that the stainless steel mesh (size 300 and 2300) alone could not separate the oil/water mixture well as the oil and water passed through the mesh unobstructively [18].

A similar study by Ifelebuegu et al. using waste cigarette butt in an oil spill clean-up found that waste filters adsorbed 16 to 26 times their weight in various oils, which is a better oil sorption performance than those commercial adsorbents. It also reported that the sorption capacity did not significantly deteriorate after 20 cycles of reuse, with up to 75% sorption capacity retained [44]. Nair reported that the highest absorption of dye using the CA membrane prepared from cigarette butts was obtained in slightly acidic conditions with the pH of 6.15 [45].

The finding suggests the effectiveness of the developed CA membrane to separate oil droplets. The good separation can be ascribed from the relatively large difference between the mean flow pore size of 0.17 µm and most of the oil droplets >0.25 µm. Those differences allow the separation through size exclusion mechanisms, in which oil droplets were retained on the top of the membrane surface [35,46].

### 3.9. Membrane Fouling Analysis

**Figure 10:**
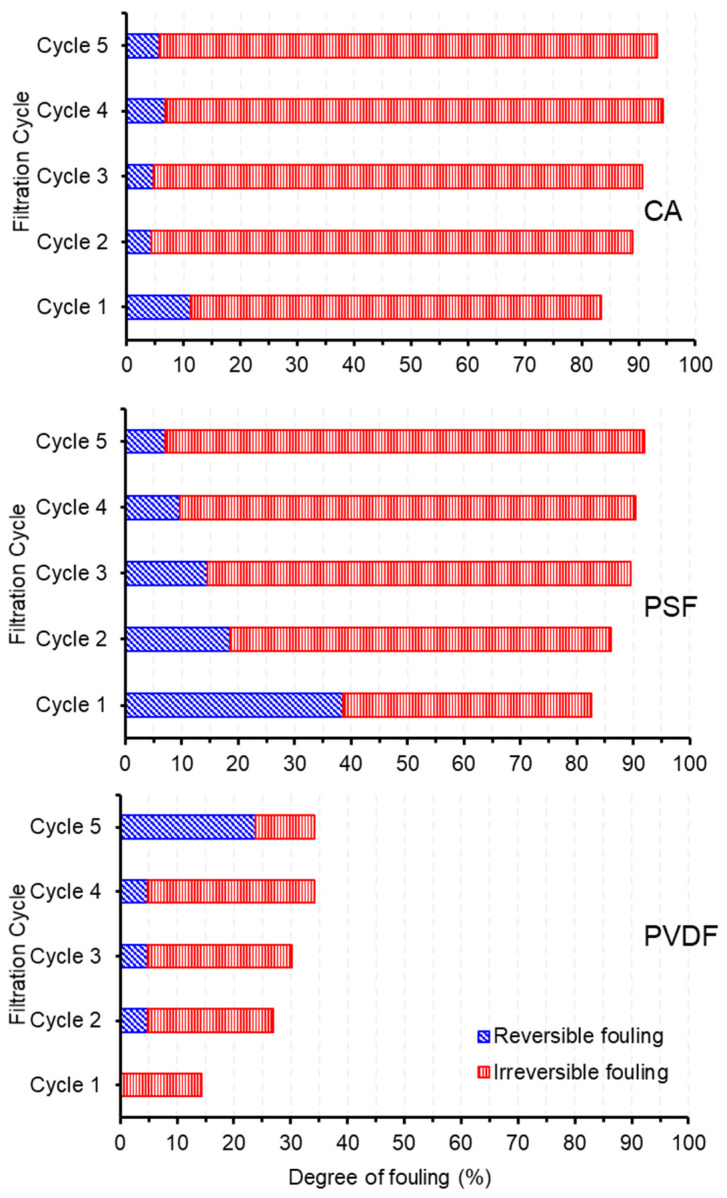
The evolution of membrane fouling in terms of reversible and irreversible fouling.

Figure 10 shows the analysis of membrane fouling based on its reversibility for CA compared to the PSF and the PVDF membranes. As expected, the total fouling for all three types of membranes showed an increasing trend with the increasing filtration cycles. The trend of multiple cycle performance is consistent with our earlier report treating the same feed following similar filtration cycles [10,11,12,47]. The three membranes pose quite distinct fouling reversibility. The total fouling depicted by PVDF at each cycle is relatively lower than the PSF and the CA membranes, indicating a lower degree of permeability loss and better antifouling properties. However, when judging from the actual permeability data in Figure 8, the performance of the PVDF membrane is comparable with the CA membrane. The low degree of fouling in the PVDF membrane compared to others is due to its relatively low clean water permeability compared to others (Figure 7). Therefore, the fouling parameters become low since the oil/water emulsion permeability was compared to the initial clean water permeability (Equations (3)–(5)). On the contrary, both CA and PSF demonstrated high total fouling since they pose high clean water permeability accompanied by similar oil/water emulsion permeability.

It is observed from Figure 10 that the membrane fouling in CA and PSF are dominated by irreversible fouling. The CA suffers a relatively high degree of irreversible fouling since the first filtration cycle. It should also be noted that CA has five-folds higher clean water permeability than PSF and PVDF at the initial cycle. It is speculated that the high fouling rate of CA was caused by the rapid compaction of permanent foulant trapped in the pores that occurred during the first cycle resulting in a lower oil/water emulsion permeability. After the first cycle, the rate of foulant accumulation is very small, indicating that the foulant was well consolidated. It is worth noting that the occurrence of membrane fouling can be well managed by implementing membrane cleaning cycles. Under proper fouling management, the lifespan of a membrane can be over 15 years [48].

The finding on a high degree of irreversible membrane fouling during the early stage of filtration indicates the possibility of further developing the phase inverted cigarette butt-based CA membrane, focusing on combating the irreversible fouling. As demonstrated in an earlier report, the incorporation of zirconia (ZrO_2_) particles in the CA casting solution resulted in a decrease in fouling resistance. The total fouling resistance for pure the CA membrane is 7.19 × 10^10^ m^−1^. The addition of 7 wt% of ZrO_2_ decreased the total fouling resistance to 2.58 × 10^10^ m^−1^ [49]. This may due to the increase in hydrophilicity of the CA membrane, which increases the interaction of the molecules on the membrane surface. A recent study reported that incorporation of cupric acetate in the non-solvent bath facilitated between the polymer with Cu that enhanced wettability, decreased surface roughness and clean water permeability [50]. The membrane properties can also be tuned through covalent functionalization of the polymer, which not only improves membrane separation properties but also the chemical and physical properties of newly synthesized materials [51].

## 4. Conclusions

This study unravels the potential of CA from cigarette butt waste as material for membrane fabrication for the oil/water emulsion treatment. This utilization of waste can alleviate the environmental problems from cigarette butt waste as well tackle the issue of oil/water emulsion. The CA-based membrane was successfully fabricated via the phase inversion method with a typical structure formed from the instantaneous demixing process. The findings show that the CA membrane poses hydrophilicity properties with a contact angle of 74.5°, lower than both PVDF and PSF membranes used as a reference. The pore size and distribution are suitable for oil/water separation. Despite being prepared from a waste cigarette, CA also poses a good surface property similar to the ones prepared from commercial PVDF and PSF polymers with equally asymmetric morphology. The pore size of CA demonstrates that the CA is within the microfiltration range. The developed CA membrane shows a promising flux of 180 L m^−2^ h^−1^ after multiple filtration cycles of oil/water emulsion. However, it still suffers a high degree of irreversible fouling (>90.0%), suggesting the potential for future improvement through optimization of fabrication parameters or via surface modification. Overall, the results demonstrate a sustainable approach in handling the oil/water emulsion pollution issue by treatment using the CA membrane derived from cigarette butt waste.

## Figures and Tables

**Table 1:** Summary of materials and weight percentage in membranes evaluated in this study.

Membrane	Polymer	Solvent	Additives	Support
CA	10 wt% of CA	90 wt% of DMF	-	Stainless steel mesh
PSF	18 wt% of PSF	80.9 wt% of DMAc	1 wt% of PEG and 0.1 wt% of LiCl	Non-woven support
PVDF	15 wt% of PVDF	85 wt% of DMAc	-	Non-woven support

CA: Cellulose acetate; PSF: Polysulfone; PVDF: Polyvinylidene difluoride; DMF: Dimethylformamide; DMAc: Dimethylacetamide; PEG: Polyethylene glycol.

**Table 2:** The elemental composition of the cellulose acetate (CA), polysulfone (PSF), and polyvinylidene difluoride (PVDF) membranes.

Membrane		Composition (%)		
	C	F	O	S
CA	51.60	0.00	48.20	0.00
PSF	69.02	0.00	26.05	4.92
PVDF	55.55	42.84	1.61	0.00

## Data Availability

Not applicable.
